# Real-World Outcomes of CDK4/6 Inhibitors in Germline BRCA1/2-Mutated Hormone Receptor-Positive, HER2-Negative Metastatic Breast Cancer: Turkish Oncology Group (TOG) Study

**DOI:** 10.3390/curroncol33060365

**Published:** 2026-06-17

**Authors:** Mustafa Seyyar, Ali Kalem, Mürsel Sali, Berkan Karabuğa, Taha Koray Sahin, Ahmet Kürşad Dişli, Alper Türkel, Berkan Karadurmuş, Ece Şahin Hafızoğlu, Nilüfer Avcı, Irem Bilgetekin, Naziyet Köse Baytemur, Esma Uguztemur, Utku Oflazoğlu, Hasibe Bilge Gür, İlhan Hacıbekiroğlu, Aysun Fatma Akkuş, Sernaz Topaloğlu, Ayberk Bayramgil, Özgecan Dülgar Kaya, Melike Yazıcı, Teoman Şakalar, Seval Akay, Nargiz Majidova, Murad Guliyev, Özkan Alan, Serkan Gülcü, Tülay Eren, Gökşen İnanç İmamoğlu, Ali Kaan Güren, Osman Köstek, Ahmet Ünlü, Banu Ozturk, Esra Aydın, Shamkhal Safarov, Bekir Doğan, Mehmet Akif Tükenmez, Teyfik Demir, Elif Şahin, Engin Erdemoğlu, Fatma Keskin Uzundere, Osman Bütün, Bülent Karabulut, Mehmet Uzun, Tuba Baydaş, Elanur Karaman, Hacı Arak, Ferhat Ekinci, Musa Barış Aykan, İsmail Ertürk, Deniz Can Guven, Adem Deligönül, Cengiz Karaçin, Öztürk Ateş, Mevlüde İnanç, Havva Yeşil, Sercan Aksoy, Tolga Köşeci, İlker Nihat Ökten, Hasan Çağrı Yıldırım, Devrim Çabuk

**Affiliations:** 1 Department of Medical Oncology, Gaziantep City Hospital, Gaziantep 27470, Türkiye; 2 Department of Medical Oncology, Faculty of Medicine, Gaziantep University, Gaziantep 27310, Türkiye; 3 Department of Medical Oncology, Faculty of Medicine, Bursa Uludağ University, Bursa 16059, Türkiye; 4 Department of Medical Oncology, Dr. Abdurrahman Yurtaslan Oncology Training and Research Hospital, Ankara 06200, Türkiye; 5 Department of Medical Oncology, Cancer Institute, Hacettepe University, Ankara 06230, Türkiye; 6 Department of Medical Oncology, Faculty of Medicine, Erciyes University, Kayseri 38280, Türkiye; 7 Department of Medical Oncology, Eskişehir City Hospital, Eskişehir 26080, Türkiye; 8 Department of Medical Oncology, Gulhane Training and Research Hospital, Ankara 06010, Türkiye; 9 Department of Medical Oncology, Faculty of Medicine, Celal Bayar University, Manisa 45030, Türkiye; ecesahin109@gmail.com (E.Ş.H.);; 10 Department of Medical Oncology, School of Health Sciences Radiotherapy Program, Fenerbahçe University, Istanbul 34758, Türkiye; 11 Department of Medical Oncology, Ankara Memorial Hospital, Ankara 06520, Türkiye; 12 Department of Medical Oncology, Adıyaman Training and Research Hospital, Adiyaman 02040, Türkiye; esmauguztemur@hotmail.com; 13 Department of Medical Oncology, Ataturk Training and Research Hospital, Izmir Katip Celebi University, Izmir 35360, Türkiye; 14 Department of Medical Oncology, Sakarya Training and Research Hospital, Sakarya 54100, Türkiye; 15 Department of Medical Oncology, Faculty of Medicine, Trakya University, Edirne 22030, Türkiye; aysunfatmadogan@gmail.com (A.F.A.); sernaz.uzunoglu@gmail.com (S.T.); 16 Department of Medical Oncology, SBU Umraniye Training and Research Hospital, Istanbul 34764, Türkiye; 17 Department of Medical Oncology, Faculty of Medicine, Kocaeli University, Kocaeli 41001, Türkiye; 18 Department of Medical Oncology, HG Hospital, Kahramanmaraş 46050, Türkiye; 19 Department of Medical Oncology, Izmir City Hospital, Izmir 35540, Türkiye; drsevalakay@hotmail.com; 20 Department of Medical Oncology, VM Medical Park Maltepe Hospital, Istanbul 34843, Türkiye; 21 Department of Medical Oncology, Cerrahpaşa Faculty of Medicine, Istanbul University-Cerrahpaşa, Istanbul 34098, Türkiye; 22 Department of Medical Oncology, Ankara Etlik City Hospital, Ankara 06170, Türkiye; 23 Department of Medical Oncology, Marmara University Pendik Training and Research Hospital, Istanbul 34899, Türkiye; 24 Department of Medical Oncology, Antalya Training and Research Hospital, Antalya 07100, Türkiye; 25 Department of Medical Oncology, Recep Tayyip Erdoğan University Training and Research Hospital, Rize 53100, Türkiye; 26 Department of Medical Oncology, Başakşehir Çam and Sakura City Hospital, Istanbul 34480, Türkiye; 27 Department of Medical Oncology, Bakırköy Dr. Sadi Konuk Training and Research Hospital, Istanbul 34147, Türkiye; 28 Department of Medical Oncology, Trabzon University Kanuni Training and Research Hospital, Trabzon 61250, Türkiye; 29 Department of Medical Oncology, Faculty of Medicine, Ondokuz Mayıs University, Samsun 55139, Türkiye; 30 Department of Medical Oncology, Kocaeli City Hospital, Kocaeli 41060, Türkiye; 31 Department of Medical Oncology, Medical Park Istanbul Oncology Hospital, Istanbul 34303, Türkiye; 32 Department of Medical Oncology, Faculty of Medicine, Dicle University, Diyarbakır 21280, Türkiye; 33 Department of Medical Oncology, Acıbadem Kent Hospital, Izmir 35630, Türkiye; 34 Department of Medical Oncology, SBU Tepecik Training and Research Hospital, Izmir 35020, Türkiye; 35 Department of Medical Oncology, Istanbul Medeniyet University Göztepe City Hospital, Istanbul 34722, Türkiye; 36 Department of Medical Oncology, Faculty of Medicine, Karadeniz Technical University, Trabzon 61080, Türkiye; 37 Department of Medical Oncology, Faculty of Medicine, Cukurova University Balcalı Hospital, Adana 01330, Türkiye; 38 Department of Medical Oncology, Faculty of Medicine, Ege University, Izmir 35100, Türkiye

**Keywords:** BRCA mutation, CDK4/6 inhibitors, hormone receptor-positive, metastatic breast cancer, palbociclib, real-world study, ribociclib

## Abstract

Germline BRCA1/2-mutated (gBRCAm) hormone receptor-positive/HER2-negative (HR+/HER2-) metastatic breast cancer (MBC) is a biologically distinct subset in which the efficacy of cyclin-dependent kinase 4/6 (CDK4/6) inhibitors remains incompletely characterized. We evaluated real-world outcomes and prognostic factors in a multicenter retrospective Turkish cohort treated with a CDK4/6 inhibitor plus endocrine therapy (June 2020–September 2025). Progression-free survival (PFS) and overall survival (OS) were estimated using Kaplan–Meier and Cox methods. Among 121 patients, 30 (24.8%) had BRCA1, 88 (72.7%) had BRCA2, and three (2.5%) had dual mutations; 66.9% received first-line therapy, with ribociclib in 69.4% and palbociclib in 29.8%. Objective response rate was 69.4% and the clinical benefit rate was 82.6%. Median PFS was 17.0 months and OS 47.0 months. PFS was numerically longer in BRCA1 than in BRCA2 carriers (25.0 vs. 14.0 months), although the difference was not statistically significant in the pairwise comparison (HR 1.50, 95% CI 0.88–2.56; log-rank *p* = 0.135); the dual BRCA1/2 subgroup (*n* = 3) had the poorest outcomes and was assessed descriptively. OS did not differ significantly between BRCA1 and BRCA2 carriers (57.0 vs. 49.0 months; log-rank *p* = 0.520). PFS did not differ between ribociclib and palbociclib (*p* = 0.192); OS favored ribociclib at borderline significance (*p* = 0.050), but this was not confirmed in Cox regression. In multivariable analysis, ECOG ≥ 1 (HR 1.85; *p* = 0.010) and fulvestrant-based therapy (HR 1.74; *p* = 0.041) predicted shorter PFS; fulvestrant also predicted worse OS (HR 2.39; *p* = 0.008). CDK4/6 inhibitor-based therapy shows meaningful activity in gBRCAm HR+/HER2- MBC; the numerically poorer outcomes observed in BRCA2 carriers are hypothesis-generating and warrant validation in larger cohorts.

## Simple Summary

Hormone receptor-positive, HER2-negative metastatic breast cancer is the most common form of advanced breast cancer, and CDK4/6 inhibitors combined with endocrine therapy have become the standard first-line treatment. A subset of patients carry inherited mutations in the BRCA1 or BRCA2 genes, which impair DNA damage repair and may alter how tumors respond to therapy. Whether CDK4/6 inhibitors are equally effective in this molecularly distinct subgroup is still unclear, as pivotal trials included few BRCA-mutated patients. In this multicenter Turkish cohort of 121 patients, CDK4/6 inhibitor-based therapy provided meaningful clinical benefits. Outcomes appeared to differ between BRCA1 and BRCA2 carriers, with BRCA1 carriers showing numerically longer progression-free survival, although this difference did not reach statistical significance. These findings suggest that the BRCA mutation subtype may be a clinically relevant factor and support further research on biomarker-driven treatment selection and the optimal sequencing of CDK4/6 inhibitors and PARP inhibitors in this population.

## 1. Introduction

Breast cancer remains the most frequently diagnosed malignancy and the leading cause of cancer-related mortality among women worldwide, with an estimated 2.3 million new cases and 685,000 deaths in 2020 [1]. Hormone receptor-positive (HR+), human epidermal growth factor receptor 2-negative (HER2-) breast cancer represents approximately 70% of all breast cancer cases and is characterized by the expression of an estrogen receptor (ER) and/or progesterone receptor (PR) in the absence of HER2 amplification or overexpression.

Germline mutations in BRCA1 and BRCA2 (gBRCAm) occur in approximately 5–10% of breast cancer patients [2]. BRCA proteins play essential roles in homologous recombination repair of DNA double-strand breaks, and their loss leads to genomic instability and increased sensitivity to DNA-damaging agents and poly (ADP-ribose) polymerase (PARP) inhibitors [3]. Although BRCA1-mutated tumors are commonly associated with triple-negative phenotypes, approximately 20–30% of BRCA1- and 70–80% of BRCA2-mutated breast cancers are HR-positive [4,5]. Consequently, a substantial proportion of gBRCAm carriers present with HR+/HER2- disease and require optimized endocrine-based strategies.

The introduction of cyclin-dependent kinase 4 and 6 (CDK4/6) inhibitors has fundamentally changed the treatment paradigm for HR+/HER2- metastatic breast cancer (MBC). Palbociclib, ribociclib, and abemaciclib have demonstrated significant improvements in progression-free survival (PFS) and overall survival (OS) when combined with endocrine therapy in both first-line and later-line settings [6,7,8]. In pivotal first-line trials, median PFS ranged from 24.8 to 28.2 months, establishing CDK4/6 inhibitor-based combinations as the standard of care. These agents inhibit CDK4 and CDK6, preventing phosphorylation of the retinoblastoma (Rb) protein and blocking cell cycle progression from G1 to S phase [9].

PARP inhibitors have demonstrated efficacy in BRCA-mutated HER2- MBC. In the OlympiAD and EMBRACA trials, olaparib and talazoparib significantly improved PFS compared with single-agent chemotherapy of the physician’s choice (capecitabine, eribulin, or vinorelbine in OlympiAD; capecitabine, eribulin, gemcitabine, or vinorelbine in EMBRACA), with a median PFS of 7.0 vs. 4.2 months and 8.6 vs. 5.6 months, respectively [10,11]. However, the optimal sequencing of PARP inhibitors and CDK4/6 inhibitors remains uncertain. Importantly, most pivotal CDK4/6 inhibitor trials either excluded or underrepresented patients with germline BRCA mutations, limiting subgroup-specific conclusions [12].

Biological interactions between DNA damage repair pathways and cell cycle regulation may influence treatment response [13]. Preclinical studies suggest that CDK4/6 inhibition can affect homologous recombination repair capacity, raising questions regarding potential synergy or resistance mechanisms in BRCA-mutated tumors [14]. Furthermore, BRCA-mutated cancers may demonstrate distinct biological characteristics, including higher proliferative activity and differential endocrine sensitivity, which could modify CDK4/6 inhibitor benefits [15].

Several mechanisms have been proposed to explain potentially reduced CDK4/6 inhibitor efficacy in this subgroup. Safonov et al. reported the enrichment of RB1 alterations among HR+/HER2- tumors in gBRCA2 carriers, suggesting that co-loss of BRCA2 and RB1 (both located on chromosome 13q) may predispose to resistance by allowing CDK4/6 inhibition to preferentially select for and expand RB1-deficient tumor cell clones during treatment [16]. In a recent landmark integrated clinicogenomic analysis of more than 5800 patients, Safonov et al. confirmed that gBRCA2 carriers experience significantly shorter PFS on CDK4/6 inhibitor plus endocrine therapy compared with germline wild-type patients (median 9.0 vs. 15.6 months; HR 2.17; *p* < 0.00001), and identified RB1 hemizygosity together with ongoing homologous recombination deficiency as key drivers of resistance [17]. Frenel et al. described higher cumulative incidence of ESR1 mutation emergence during first-line aromatase inhibitor plus palbociclib therapy in BRCA1/2-PALB2 mutation carriers [18]. In addition, Rodriguez et al. observed a high prevalence of non-luminal intrinsic subtypes in gBRCA2-associated HR+/HER2- tumors, which have been associated with inferior CDK4/6 inhibitor outcomes [19,20].

Despite these biological considerations and the widespread use of CDK4/6 inhibitors in clinical practice, real-world evidence specifically evaluating their effectiveness in BRCA-mutated HR+/HER2- MBC remains limited and heterogeneous [21]. Many published studies include small numbers of gBRCAm patients, and methodological issues such as immortal time bias and inconsistent control group definitions have been highlighted [22,23]. Current NCCN and ESMO guidelines recommend CDK4/6 inhibitors as the preferred first-line therapy for HR+/HER2- MBC, irrespective of BRCA mutation status, largely extrapolated from trials not specifically designed to evaluate this molecular subgroup.

Against this background of biological complexity and limited subgroup-specific clinical data, this study aimed to evaluate real-world clinical outcomes of CDK4/6 inhibitor-based therapy in patients with germline BRCA1/2 mutations and HR+/HER2- metastatic breast cancer, including PFS, OS, response rates, safety profile, and independent prognostic factors, with particular attention to differences between BRCA1 and BRCA2 mutation carriers.

## 2. Materials and Methods

### 2.1. Study Design and Patient Selection

This retrospective, multicenter cohort study included patients with pathogenic germline BRCA1 or BRCA2 mutations who received CDK4/6 inhibitor-based therapy for HR+/HER2- metastatic breast cancer between June 2020 and September 2025 at participating centers in Turkey. Germline BRCA1/2 testing was performed at the time of initial breast cancer diagnosis using next-generation sequencing-based hereditary cancer panels at certified clinical laboratories, and therefore preceded the initiation of CDK4/6 inhibitor therapy for metastatic disease. Variants were classified as pathogenic or likely pathogenic according to American College of Medical Genetics and Genomics (ACMG) criteria; variants of uncertain significance were not included. This research obtained ethical approval from the Gaziantep City Hospital Ethics Committee (Project code: 244/2025). The study was approved by the institutional review boards of all participating centers and conducted in accordance with the Declaration of Helsinki.

Inclusion criteria comprised: (1) confirmed pathogenic germline BRCA1 or BRCA2 mutation by validated genetic testing; (2) histologically confirmed breast carcinoma; (3) HR+ (ER ≥ 10% by immunohistochemistry) and HER2- disease (according to ASCO/CAP guidelines); (4) metastatic disease (Stage IV) at the time of CDK4/6 inhibitor initiation; (5) treatment with at least one dose of a CDK4/6 inhibitor (palbociclib, ribociclib, or abemaciclib) in combination with endocrine therapy; (6) age ≥ 18 years at treatment initiation; and (7) adequate baseline clinical and radiological data available for assessment. Exclusion criteria included: (1) the presence of other concurrent malignancies; (2) insufficient follow-up data (<1 imaging assessment); and (3) the use of CDK4/6 inhibitors outside of standard-of-care indications. During the study period, abemaciclib was administered to only one patient, primarily owing to national reimbursement conditions under which ribociclib and palbociclib were the predominantly accessible CDK4/6 inhibitors. This patient was included in the overall cohort but not in the agent-specific comparisons, which were restricted to ribociclib versus palbociclib.

### 2.2. Data Collection

Clinical and demographic data were extracted from electronic medical records, including demographic characteristics (age, menopausal status, ECOG performance status), tumor characteristics (histological subtype, tumor grade, ER/PR expression, Ki-67 proliferation index, sites of metastatic disease), genetic characteristics (BRCA mutation type), treatment details (CDK4/6 inhibitor agent, endocrine therapy partner, line of therapy, treatment duration, dose modifications), outcomes (best response, progression dates, survival status), and adverse events (type, grade per CTCAE v5.0). Tumor responses were assessed by the treating physician based on radiological evaluations performed during routine clinical practice, applying RECIST 1.1 criteria, and categorized as complete response (CR), partial response (PR), stable disease (SD), or progressive disease (PD). The objective response rate (ORR) was defined as CR + PR, and clinical benefit rate (CBR) as CR + PR + SD.

### 2.3. Study Endpoints

The primary endpoints were PFS, defined as the time from CDK4/6 inhibitor initiation to radiological or clinical disease progression or death from any cause, and OS, defined as the time from CDK4/6 inhibitor initiation to death from any cause. Secondary endpoints included objective response rate (ORR), clinical benefit rate (CBR), 24-month survival rates, and safety profile (treatment-related dose modifications and discontinuations). Pre-specified subgroup analyses included a comparison of PFS and OS according to BRCA mutation subtype (BRCA1, BRCA2, and BRCA1 + BRCA2), CDK4/6 inhibitor type (ribociclib versus palbociclib), and line of therapy.

### 2.4. Statistical Analysis

Descriptive statistics were used to summarize patient characteristics. Categorical variables were expressed as frequencies and percentages and continuous variables as medians with interquartile ranges. Survival analyses were performed using the Kaplan–Meier method. PFS and OS were estimated with 95% confidence intervals (CIs). Comparisons between subgroups were performed using the log-rank test for survival outcomes and Fisher’s exact test or chi-square test for categorical variables. Because only three patients carried concurrent BRCA1 and BRCA2 mutations, the primary survival comparison was restricted to BRCA1 versus BRCA2 carriers using the log-rank test; this dual-mutation subgroup was summarized descriptively and was not included in formal between-group testing. To evaluate whether the association between BRCA subtype and survival was confounded by line of therapy, we additionally performed line-stratified Kaplan–Meier analyses, tested the BRCA mutation type × line-of-therapy interaction in a Cox proportional hazards model, and estimated the BRCA2-versus-BRCA1 hazard ratio after adjusting for line of therapy. Univariable Cox proportional hazards regression analyses were conducted to assess the association between baseline variables and PFS or OS. Variables with a *p*-value < 0.10 in univariable analysis and/or with established clinical relevance were included in multivariable Cox regression models. Results are presented as hazard ratios (HRs) with 95% CIs. No imputation was performed for missing data; analyses were conducted on available cases for each variable. All statistical tests were two-sided, and *p*-values < 0.05 were considered statistically significant. Statistical analyses were performed using SPSS version 28.0 (IBM Corp., Armonk, NY, USA).

## 3. Results

### 3.1. Patient Characteristics

A total of 121 patients with HR+/HER2- metastatic breast cancer harboring pathogenic germline BRCA mutations were included. Among these, 30 (24.8%) had BRCA1 mutations, 88 (72.7%) had BRCA2 mutations, and three (2.5%) carried concurrent BRCA1 and BRCA2 mutations.

Baseline clinicopathologic characteristics are summarized in Table 1. The majority of patients were female (94.2%), premenopausal (57.0%), and had an ECOG performance status of 0 (58.7%). Most tumors were ductal histology (93.4%) and grade 2 (65.3%). Bone was the most common site of metastatic involvement (71.9%), followed by visceral (43.8%), lung (24.8%), liver (20.7%), and central nervous system (5.8%) metastases. CDK4/6 inhibitors were administered in the first-line setting in 66.9% of cases. Ribociclib was used in 69.4%, palbociclib in 29.8%, and abemaciclib in 0.8% of patients. The endocrine therapy partner was letrozole in 69.4% and fulvestrant in 30.6% of cases. There were no statistically significant baseline differences between BRCA1 and BRCA2 groups.

### 3.2. Efficacy, Survival and Safety Outcomes

The overall response rate was 69.4% and the clinical benefit rate was 82.6%. Median PFS for the entire cohort was 17.0 months and median OS was 47.0 months. Dose reduction occurred in 16.5% and treatment discontinuation in 2.5% of patients. The efficacy, survival and safety outcomes stratified by BRCA mutation type are summarized in Table 2.

**Figure 1:**
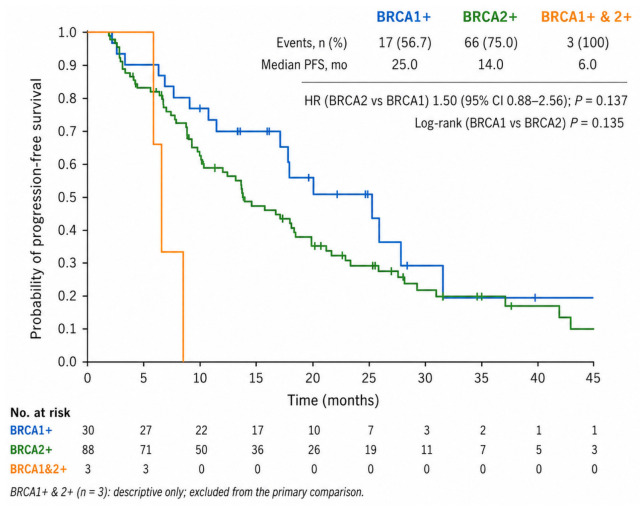
Kaplan–Meier curve for PFS according to BRCA status. Footnote: PFS was estimated using the Kaplan–Meier method and compared between BRCA1 and BRCA2 using the log-rank test. Hazard ratios and 95% confidence intervals were derived from Cox proportional hazards regression, with BRCA1 as the reference group. The concurrent BRCA1+/BRCA2+ subgroup included a very small number of patients and is presented descriptively; results for this subgroup should be interpreted with caution.

**Figure 2:**
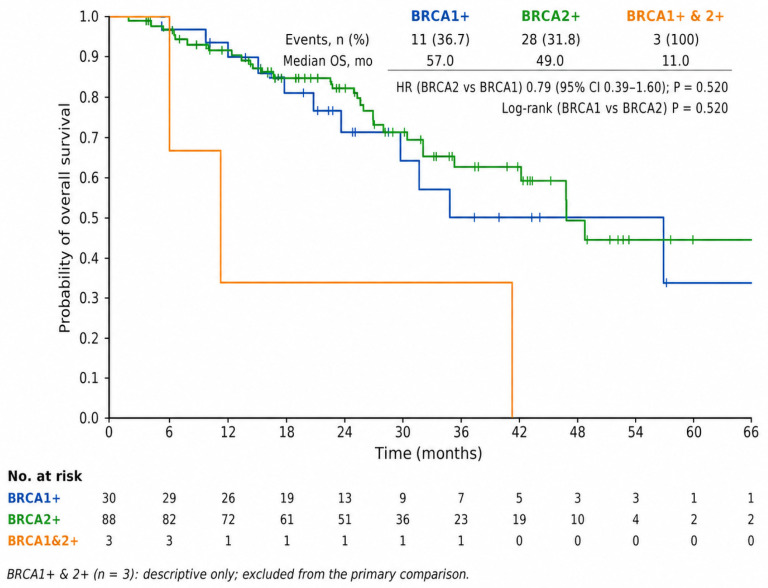
Kaplan–Meier curve for OS according to BRCA status. Footnote: OS was estimated using the Kaplan–Meier method and compared using the log-rank test. Hazard ratios and 95% confidence intervals were calculated using univariable Cox proportional hazards regression. BRCA1+ was used as the reference category. The BRCA1+/BRCA2+ subgroup included a very small number of patients and should be interpreted with caution.

When stratified by BRCA mutation type, BRCA1 carriers had a numerically longer median PFS than BRCA2 carriers (25.0 vs. 14.0 months); however, this difference was not statistically significant in the primary pairwise comparison (log-rank *p* = 0.135; Cox HR for BRCA2 vs. BRCA1, 1.50, 95% CI 0.88–2.56; *p* = 0.137; Figure 1). The three patients with concurrent BRCA1 and BRCA2 mutations had the poorest outcomes (median PFS 6.0 months); owing to the very small sample size (*n* = 3), this subgroup was assessed descriptively and was excluded from the primary comparison. For OS, median survival was numerically higher in BRCA1 than in BRCA2 carriers (57.0 vs. 49.0 months), but the difference was not statistically significant (log-rank *p* = 0.520; Figure 2). Safety outcomes were comparable between the BRCA1 and BRCA2 groups.

**Figure 3:**
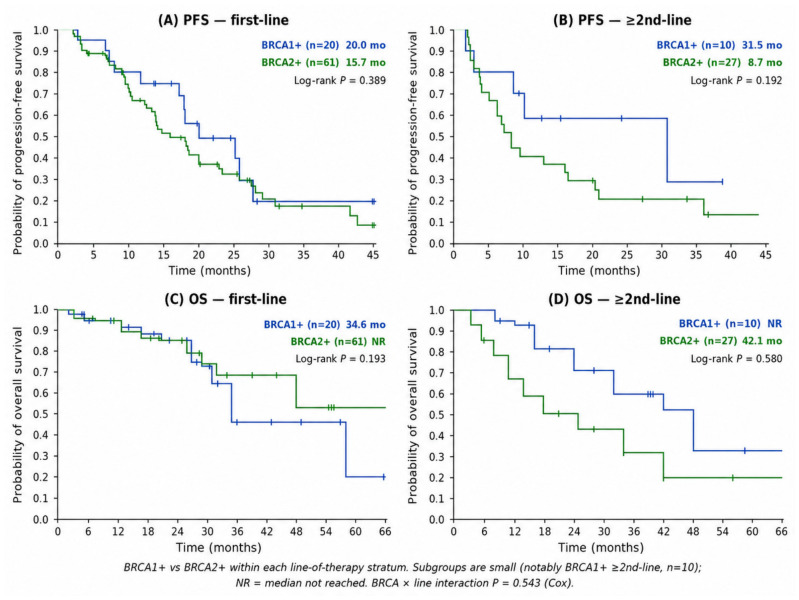
PFS and OS in BRCA1 versus BRCA2 carriers, stratified by line of therapy. (**A**) PFS in the first-line setting. (**B**) PFS in the ≥2nd-line setting. (**C**) OS in the first-line setting. (**D**) OS in the ≥2nd-line setting. Footnote: PFS and OS were estimated using the Kaplan–Meier method and compared between BRCA1 and BRCA2 carriers using the log-rank test within each line-of-therapy stratum. Median values and log-rank *p*-values are shown in each panel; NR denotes median not reached. Subgroup sizes were limited, particularly for BRCA1 carriers treated in the ≥2nd-line setting (*n* = 10).

PFS and OS were also compared between BRCA1 and BRCA2 carriers within each line-of-therapy stratum (Figure 3). In the first-line setting, median PFS was 20.0 months for BRCA1 versus 15.7 months for BRCA2 carriers (log-rank *p* = 0.389), and median OS was 34.6 months versus not reached (log-rank *p* = 0.193). In the ≥2nd-line setting, median PFS was 31.5 versus 8.7 months (log-rank *p* = 0.192), and median OS was not reached versus 42.1 months (log-rank *p* = 0.580). The interaction between BRCA mutation type and line of therapy was not significant (Cox interaction term, *p* = 0.543); after adjustment for line of therapy, the BRCA2-versus-BRCA1 hazard ratio for PFS was 1.51 (95% CI 0.89–2.58; *p* = 0.13).

**Figure 4:**
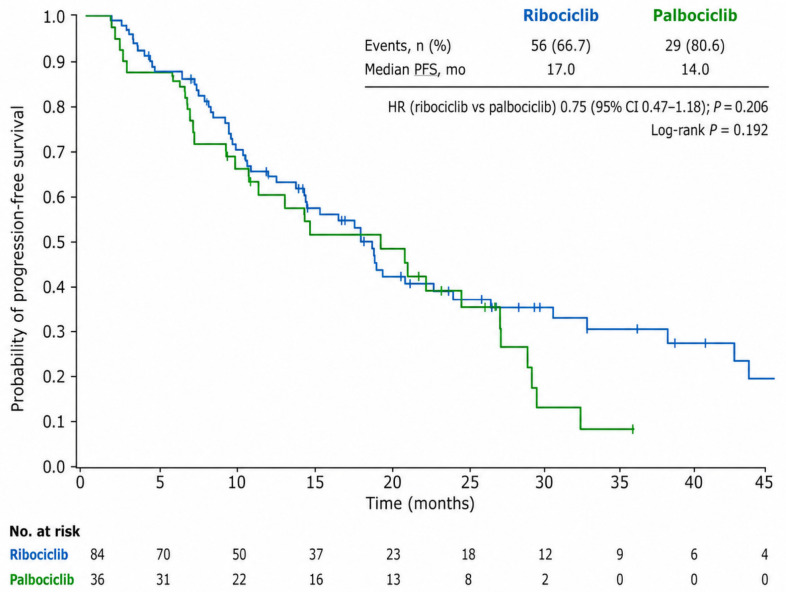
Kaplan–Meier curve for PFS according to CDK4/6 inhibitor. Footnote: PFS was estimated using the Kaplan–Meier method and compared using the log-rank test. Hazard ratios and 95% confidence intervals were calculated using univariable Cox proportional hazards regression, with palbociclib as the reference group.

**Figure 5:**
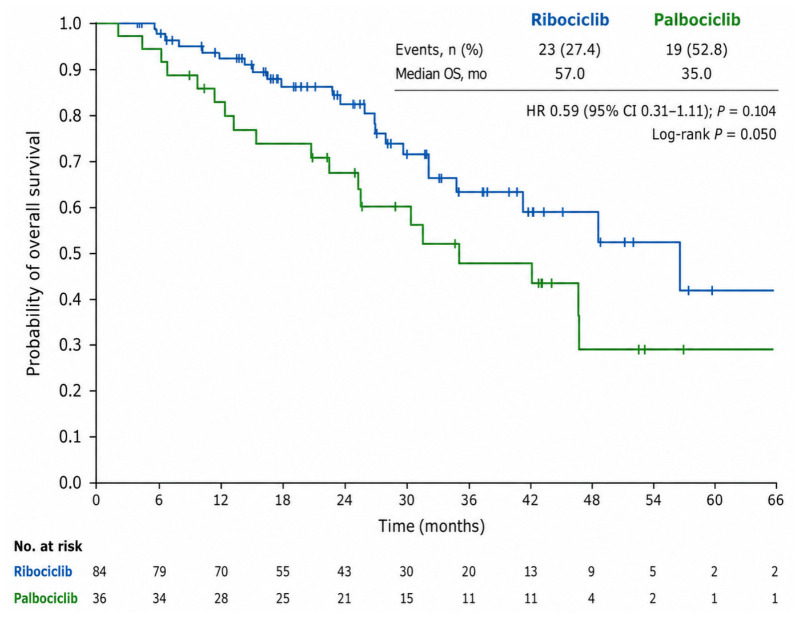
Kaplan–Meier curve for OS according to CDK4/6 inhibitor. Footnote: Overall survival was estimated using the Kaplan–Meier method and compared using the log-rank test. Hazard ratios and 95% confidence intervals were obtained from univariable Cox proportional hazards regression, with palbociclib as the reference group.

When stratified by CDK4/6 inhibitor, median PFS was numerically longer with ribociclib than with palbociclib; however, this difference was not statistically significant (log-rank *p* = 0.192; Figure 4). For OS, Kaplan–Meier curves demonstrated separation favoring ribociclib (log-rank *p* = 0.050; Figure 5). In univariable Cox analysis, ribociclib was associated with a numerically lower risk of death, though not statistically significant. Ribociclib was the most frequently used CDK4/6 inhibitor in both subgroups, with similar proportions among BRCA1 (73.3%) and BRCA2 (69.3%) carriers.

**Figure 6:**
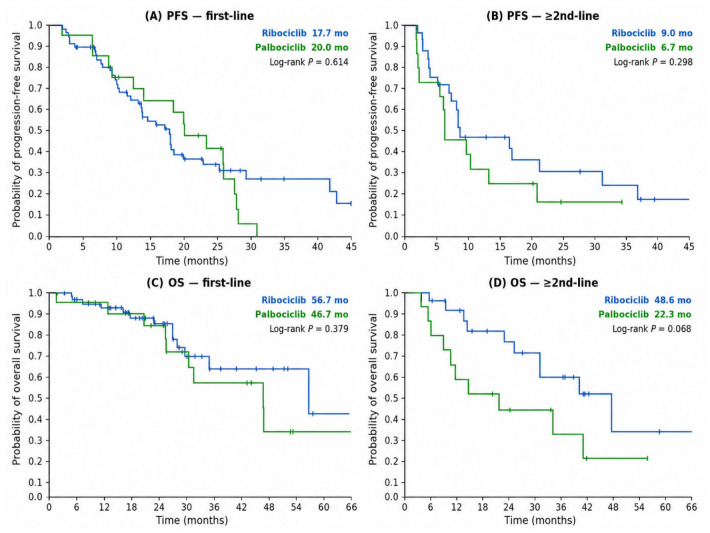
PFS and OS according to CDK4/6 inhibitor type and line of therapy. (**A**) PFS in the first-line setting. (**B**) PFS in patients treated in the ≥2nd-line setting. (**C**) OS in the first-line setting. (**D**) OS in patients treated in the ≥2nd-line setting. Footnote: PFS and OS were estimated using the Kaplan–Meier method and compared between ribociclib- and palbociclib-treated patients using the log-rank test, separately within the first-line and ≥2nd-line settings. Median values and log-rank *p*-values are shown in each panel.

Survival outcomes were compared according to the treatment line in which CDK4/6 inhibitors were administered (Figure 6). In the first-line setting, no significant differences were observed between ribociclib and palbociclib in either PFS (median 18 vs. 20 months; log-rank *p* = 0.614; Figure 6A) or OS (median 57 vs. 47 months; log-rank *p* = 0.379; Figure 6C). In the ≥2nd-line setting, ribociclib showed numerically longer PFS (median 9 vs. 7 months; log-rank *p* = 0.298; Figure 6B) and OS (median 49 vs. 22 months; log-rank *p* = 0.068; Figure 6D); however, differences did not reach statistical significance.

### 3.3. Univariable and Multivariable Analysis: Independent Predictors of PFS and OS

Univariable and multivariable Cox regression analyses for PFS and OS are summarized in Table 3 and Table 4. For PFS, ECOG performance status ≥ 1 (HR 1.85, 95% CI 1.16–2.94; *p* = 0.010) and fulvestrant-based endocrine therapy (HR 1.74, 95% CI 1.02–2.94; *p* = 0.041) were independently associated with shorter progression-free survival in multivariable analysis. For OS, endocrine partner remained the only independent prognostic factor: fulvestrant-based therapy was associated with inferior OS compared with letrozole-based therapy (HR 2.39, 95% CI 1.26–4.54; *p* = 0.008). ECOG performance status and CDK4/6 inhibitor type were not independently associated with OS.

## 4. Discussion

This multicenter retrospective study represents one of the largest real-world cohorts evaluating CDK4/6 inhibitor-based therapy specifically in patients with germline BRCA1/2-mutated HR+/HER2- MBC. In the overall population, median PFS was 17.0 months and median OS was 47.0 months. Numerically, BRCA1 carriers had a longer median PFS (25.0 vs. 14.0 months) and median OS (57.0 vs. 49.0 months) than BRCA2 carriers; however, neither difference reached statistical significance (log-rank *p* = 0.135; OS *p* = 0.520). The numerically longer PFS in BRCA1 carriers nonetheless raises the possibility of biological heterogeneity between BRCA1- and BRCA2-associated tumors.

Median PFS in our cohort was numerically shorter than in pivotal trials such as PALOMA-2 and MONALEESA-2 [24,25]. However, real-world populations differ substantially from trial populations in disease burden, comorbidities, treatment sequencing, and monitoring intervals. Importantly, 33.1% of patients in our study received CDK4/6 inhibitors in second or later lines, which likely contributed to shorter overall PFS. When restricted to first-line treatment, median PFS was 20 months with palbociclib and 18 months with ribociclib, without significant differences. These results, though somewhat lower than trial outcomes, are consistent with the expected attenuation of benefit in heterogeneous routine-practice cohorts.

In OS analyses across the entire cohort, median OS was 57.0 months with ribociclib and 35.0 months with palbociclib (log-rank *p* = 0.050). Although ribociclib was associated with a numerically lower risk of death (HR 0.59), this did not reach statistical significance in Cox regression analysis (*p* = 0.104). In the first-line treatment, median OS was 57.0 months with ribociclib and 47.0 months with palbociclib, without a statistically significant difference (log-rank *p* = 0.379). Notably, these survival estimates appear shorter than those reported in pivotal randomized trials—63.9 months for ribociclib in MONALEESA-2 and 53.9 months for palbociclib in PALOMA-2. This discrepancy likely reflects real-world case-mix heterogeneity, inclusion of later-line patients, differential treatment sequencing, and the distinct biological features of germline BRCA-mutated tumors rather than intrinsic differences in CDK4/6 inhibitor efficacy.

The interaction between DNA damage response pathways and cell cycle regulation may further influence CDK4/6 inhibitor efficacy in BRCA-mutated tumors. BRCA deficiency results in impaired homologous recombination repair and increased genomic instability [26]. Preclinical studies suggest that the combination of CDK4/6 and PARP inhibitors produces synergistic antitumor activity, including in breast cancer models, indicating a potentially favorable interaction when these agents are used concurrently [27,28]. Mechanistically, CDK4/6 inhibition can impair homologous recombination repair—for instance, by promoting PARP1 degradation and reducing the availability of homologous recombination repair factors—which may contribute to this synergy [27]. Whether this cross-talk also influences—favorably or unfavorably—the efficacy of PARP inhibitors administered sequentially after CDK4/6 inhibition remains undefined. Separately, some evidence suggests that BRCA mutations might be associated with enhanced sensitivity to CDK4/6 inhibition through mechanisms involving E2F-mediated transcription and replication stress [26,29].

Our results align closely with the recent systematic review and meta-analysis by Bottosso et al., which demonstrated that gBRCAm patients treated with CDK4/6 inhibitors experienced significantly worse outcomes compared to gBRCA wild-type patients (PFS HR 1.68, OS HR 1.73) [22]. The meta-analysis included 14 studies covering 618 gBRCAm patients, though most were retrospective with moderate-to-high risk of bias. Our study contributes to this growing body of evidence with detailed multivariable analyses identifying independent predictors of survival and comprehensive characterization of the BRCA1 versus BRCA2 subgroups.

A notable observation in our study was the numerically longer PFS among BRCA1 than BRCA2 carriers (25.0 vs. 14.0 months); however, this difference did not reach statistical significance (log-rank *p* = 0.135; HR for BRCA2 vs. BRCA1 1.50, 95% CI 0.88–2.56), nor did the corresponding OS difference (57.0 vs. 49.0 months, *p* = 0.520). Notably, the longer PFS in BRCA1 carriers was directionally consistent across both the first-line and ≥2nd-line settings, with no significant interaction between BRCA mutation type and line of therapy, suggesting that this difference is unlikely to be explained by line of therapy alone. BRCA mutation type was not independently prognostic in multivariable models, and the numerically shorter PFS in BRCA2 carriers may be partly attributable to their more frequent use of fulvestrant-based therapy (31.8% vs. 20.0%), which was independently associated with shorter PFS. Beyond such treatment-related factors, however, compelling biological evidence supports a BRCA2-specific resistance mechanism. Safonov et al. first identified enrichment of RB1 alterations among HR+/HER2- tumors in gBRCA2 carriers, hypothesizing that co-loss of BRCA2 and RB1 (both located on chromosome 13q) could predispose to biallelic RB1 loss, as CDK4/6 inhibition preferentially selects for and expands tumor cell clones that have acquired complete RB1 loss [16,30]. In a landmark subsequent integrated clinicogenomic analysis of more than 5800 patients, the same group demonstrated that this vulnerability operates through a dual mechanism. First, baseline RB1 hemizygosity lowers the evolutionary barrier to complete RB1 loss; second, ongoing homologous recombination deficiency actively drives the acquisition variants with RB1 function-loss, which are then positively selected and clonally expanded under the pressure of CDK4/6 inhibition [17]. In that study, gBRCA2 carriers treated with an CDK4/6 inhibitor plus endocrine therapy experienced a significantly shorter PFS compared with germline wild-type patients (median 9.0 versus 15.6 months; HR 2.17; *p* < 0.00001), and patient-derived xenograft models from gBRCA2 carriers demonstrated near-uniform resistance to CDK4/6 inhibitors with consistent post-treatment Rb loss [17]. Additionally, Rodriguez et al. reported that 63% of gBRCA2 HR+/HER2- tumors exhibited non-luminal intrinsic subtypes by PAM50, which have been associated with inferior CDK4/6 inhibitor outcomes [19,20]. These convergent lines of evidence require further validation through comprehensive genomic analyses integrating RB1 allele-specific copy number status, intrinsic subtype, and homologous recombination deficiency signatures in prospective cohorts.

An apparent dissociation between PFS and OS in BRCA2 carriers warrants comment. Despite their numerically shorter PFS relative to BRCA1 carriers (14.0 vs. 25.0 months), overall survival did not differ significantly between the subgroups (49.0 vs. 57.0 months; *p* = 0.520), and the 24-month OS rate was in fact numerically higher in BRCA2 carriers (82% vs. 72%). Several factors may reconcile this pattern. First, the resistance mechanism proposed by Safonov et al. [17]—RB1 hemizygosity and ongoing homologous recombination deficiency driving acquired RB1 loss—acts predominantly on CDK4/6 inhibitor efficacy and is therefore expected to manifest most directly as shortened PFS, precisely as observed. Second, OS in the BRCA2 subgroup remained immature, with only 31.8% of patients having died and an inestimable upper confidence bound for median OS, so that firm OS conclusions are premature. Third, and most importantly, OS in BRCA-mutated disease is strongly influenced by the efficacy of subsequent therapies: patients with gBRCAm have access to highly active post-progression options—particularly PARP inhibitors and platinum-based chemotherapy—and the resulting benefit may offset a shorter PFS on CDK4/6 inhibitor therapy. A shorter PFS on first-line CDK4/6 inhibition therefore does not necessarily translate into inferior survival in this population, an observation that further underscores the importance of optimal treatment sequencing. Finally, because ribociclib use and treatment line were balanced between the BRCA subgroups, these OS patterns are unlikely to reflect differential treatment allocation; the OS separation favoring ribociclib in the overall cohort was not retained in multivariable analysis and should be regarded as hypothesis-generating. Taken together, these data indicate that the unfavorable biology associated with BRCA2-mutated tumors is most evident in PFS on CDK4/6 inhibitor therapy, whereas OS is shaped by additional factors, including treatment sequencing and the availability of effective salvage therapies.

In multivariable analysis, ECOG performance status and endocrine partner were independent predictors of outcome. ECOG ≥ 1 remained significantly associated with shorter PFS, reinforcing its established prognostic role in metastatic breast cancer. The endocrine backbone also influenced survival endpoints, with fulvestrant-based combinations associated with inferior PFS and OS compared with aromatase inhibitor-based therapy. This likely reflects treatment selection bias rather than the intrinsic inferiority of fulvestrant. In routine practice, fulvestrant is frequently administered in later lines or in endocrine-resistant settings, where disease biology is more aggressive and prior endocrine exposure may reduce responsiveness [7,31]. Nevertheless, randomized trials such as MONALEESA-3 and MONARCH-2 have demonstrated the clear benefit of fulvestrant-based CDK4/6 combinations [6,32].

The shorter outcomes observed with fulvestrant in our cohort may also reflect accumulated endocrine resistance mechanisms, including ESR1 mutations and ligand-independent ER activation [18,33]. In BRCA-mutated tumors, additional genomic instability and replication stress may accelerate resistance evolution [14]. Frenel et al. reported higher cumulative incidence of ESR1 mutation emergence during first-line palbociclib therapy in BRCA1/2-PALB2 mutation carriers [18], suggesting accelerated acquisition of endocrine resistance mechanisms in this population.

Several biological mechanisms have been proposed to explain the reduced CDK4/6 inhibitor benefit in BRCA-mutated tumors beyond RB1 co-deletion. In a correlative analysis of two neoadjuvant phase II trials in early HR+/HER2- breast cancer (GIADA and LETLOB), Griguolo et al. found that a transcriptomic HRD gene-expression signature and an RB-loss gene-expression signature correlated with each other, and that higher levels of both were associated with lower sensitivity to endocrine therapy and higher sensitivity to chemotherapy-based therapy [34]. Importantly, these associations relate to gene-expression signatures and treatment response, rather than to the genomic co-occurrence of BRCA mutation and RB1 deletion. Although CDK4/6 inhibitors were not directly evaluated in that study, the RB-loss signature has been proposed as a marker of CDK4/6 inhibitor resistance; collectively, these observations support the possibility that homologous recombination deficiency may contribute to primary endocrine resistance that is incompletely overcome by CDK4/6 inhibition. The absence of comprehensive molecular profiling in our cohort limits mechanistic interpretation, highlighting the need for integrated genomic analyses in future studies.

The optimal sequencing of CDK4/6 inhibitors and PARP inhibitors in gBRCAm HR+/HER2- metastatic breast cancer remains unresolved. PARP inhibitors have demonstrated improved PFS compared with single-agent chemotherapy of the physician’s choice (capecitabine, eribulin, or vinorelbine in OlympiAD; capecitabine, eribulin, gemcitabine, or vinorelbine in EMBRACA) in these respective trials [10,11], though both included predominantly pretreated populations. However, current clinical guidelines recommend CDK4/6 inhibitors as the preferred first-line therapy irrespective of BRCA status. Our data support continued use of CDK4/6 inhibitors in this population, while underscoring the need for prospective studies to define optimal sequencing strategies. In this context, the ongoing EvoPAR-Breast01 study (NCT06380751) is directly addressing this critical question, evaluating saruparib (AZD5305), a next-generation selective PARP1 inhibitor, plus camizestrant versus the physician’s choice of CDK4/6 inhibitor and endocrine therapy as the first-line treatment in patients with HR+/HER2- MBC harboring germline or somatic alterations in selected homologous recombination deficiency genes. Results from this prospective randomized trial may provide the level I evidence needed to guide optimal first-line treatment selection in this molecularly defined subgroup.

In early stage disease, adjuvant abemaciclib and ribociclib have demonstrated benefits in high-risk HR+/HER2- populations [35,36], and adjuvant olaparib improved outcomes in gBRCAm HER2-negative disease [37]. These overlapping indications may complicate therapeutic prioritization in BRCA-mutated patients. Although our findings derive from the metastatic setting and cannot be directly extrapolated to early stage disease, the attenuated CDK4/6 inhibitor benefit observed among BRCA2 carriers raises the hypothesis that adjuvant CDK4/6 inhibitor efficacy might similarly be reduced in this subgroup—where adjuvant olaparib is also an option—a question that warrants dedicated prospective evaluation. The role and optimal sequencing of adjuvant CDK4/6 inhibitors following the completion of one year of adjuvant olaparib in BRCA carriers also remains unclear. In the metastatic setting, our findings do not suggest that CDK4/6 inhibitors should be withheld solely based on BRCA mutation status; rather, they highlight that BRCA subtype and clinical context should be carefully considered when individualizing treatment decisions.

The safety profile in our cohort was consistent with known CDK4/6 inhibitor toxicities. Dose reductions occurred in 16.5% and discontinuation in 2.5% of patients. As expected, neutropenia was the most common toxicity [24,38]. No unexpected safety signals were observed, and tolerability did not appear to differ between BRCA1 and BRCA2 carriers. These findings indicate that germline BRCA mutation status does not substantially alter the safety profile of CDK4/6 inhibitors.

This study has several strengths, including a relatively large BRCA-mutated cohort (*n* = 121), detailed subgroup analyses, and multivariable modeling of prognostic factors. However, limitations must be acknowledged. The retrospective design introduces potential selection bias and limits causal inference. The relatively small number of BRCA1 carriers (*n* = 30; 17 progression events) limited the statistical power of the BRCA1-versus-BRCA2 comparison, which was adequately powered to detect only large effects (approximately 80% power for a hazard ratio of ≥2.0); the more modest difference actually observed (HR 1.50; 95% CI 0.88–2.56) could therefore be neither confirmed nor excluded, and this subgroup comparison should be regarded as exploratory and hypothesis-generating. Additionally, the absence of a concurrent BRCA wild-type comparator group precludes direct comparison of CDK4/6 inhibitor outcomes between BRCA-mutated and non-mutated populations within our dataset; however, given that CDK4/6 inhibitor efficacy in unselected HR+/HER2- MBC has been extensively characterized across multiple large-scale phase III trials and real-world datasets, the primary aim of the present study was to characterize outcomes specifically within the BRCA-mutated subgroup and to delineate differences between BRCA1 and BRCA2 carriers. We lacked comprehensive molecular profiling data beyond BRCA mutation status, including RB1 allele-specific copy number, ESR1 mutations, PIK3CA alterations, and tumor mutational signatures that might provide additional insights into treatment resistance. In addition, germline testing was performed across multiple accredited laboratories using different assays and panels, and centralized data on sequencing depth and on the uniform application of large-rearrangement analysis (e.g., MLPA) were not available; although all patients had a pathogenic or likely pathogenic BRCA variant confirmed by an accredited laboratory, rare misclassification due to undetected structural variants cannot be entirely excluded.

### Clinical Implications and Future Directions

Our findings support CDK4/6 inhibitor-based therapy as an effective treatment option for BRCA-mutated HR+/HER2- metastatic breast cancer, though outcomes appear inferior to unselected populations. The numerically longer PFS observed in BRCA1 versus BRCA2 carriers is hypothesis-generating and warrants validation in larger cohorts. The mechanistic framework established by Safonov et al. [17], linking RB1 hemizygosity and homologous recombination deficiency to CDK4/6 inhibitor resistance specifically in gBRCA2 carriers, provides a compelling rationale for prospective biomarker-driven studies integrating pretreatment RB1 allele-specific copy number assessment alongside BRCA mutation status. Future research priorities include prospective randomized trials comparing CDK4/6 inhibitors versus PARP inhibitors in first-line therapy—a question being directly addressed by the ongoing EvoPAR-Breast01 study (NCT06380751)—evaluation of optimal treatment sequencing strategies, and identification of predictive biomarkers beyond BRCA mutation status. The integration of comprehensive genomic profiling, including ESR1 mutations, RB1 alterations, and homologous recombination deficiency scores, may enable biomarker-driven treatment selection and elucidate resistance mechanisms in this molecularly distinct population.

## 5. Conclusions

In this multicenter real-world cohort of patients with germline BRCA-mutated HR+/HER2- metastatic breast cancer, CDK4/6 inhibitor-based therapy demonstrated meaningful clinical efficacy with a median PFS of 17.0 months and median OS of 47.0 months. These outcomes are numerically lower than those observed in unselected HR+/HER2- MBC populations from pivotal trials, consistent with recent meta-analyses. BRCA1 carriers showed numerically superior outcomes compared with BRCA2 carriers; although this difference did not reach statistical significance, its direction is consistent with mechanistic evidence implicating RB1 hemizygosity and homologous recombination deficiency as drivers of CDK4/6 inhibitor resistance in gBRCA2-associated tumors. While CDK4/6 inhibitors provide valuable clinical benefit and should not be withheld from BRCA-mutated patients, these data highlight the potential value of considering BRCA subtype when individualizing treatment decisions. Prospective comparative trials, including the ongoing EvoPAR-Breast01 study, are needed to optimize treatment sequencing with PARP inhibitors and identify predictive biomarkers in this molecularly distinct population.

## Figures and Tables

**Table 1:** Baseline characteristics.

Characteristic	All Patients	BRCA1	BRCA2	BRCA1 + 2	*p*
**Age (median)**	44	45	44	37	0.186
**Sex**					
Female	114 (94.2%)	28 (93.3%)	83 (94.3%)	3 (100.0%)	1.000
Male	7 (5.8%)	2 (6.7%)	5 (5.7%)	0 (0.0%)	
**Menopausal status**					
Premenopausal	69 (57.0%)	16 (53.3%)	51 (58.0%)	2 (66.7%)	0.905
Postmenopausal	45 (37.2%)	12 (40.0%)	32 (36.4%)	1 (33.3%)	
Male	7 (5.8%)	2 (6.7%)	5 (5.7%)	0 (0.0%)	
**ECOG**					
0	71 (58.7%)	18 (60.0%)	50 (56.8%)	3 (100.0%)	0.816
≥1	50 (41.3%)	12 (40.0%)	38 (43.2%)	0 (0.0%)	
**Disease presentation**					
De novo metastatic	54 (44.6%)	13 (43.3%)	40 (45.5%)	1 (33.3%)	1.000
Recurrent	67 (55.4%)	17 (56.7%)	48 (54.5%)	2 (66.7%)	
**Histology**					
Ductal	113 (93.4%)	29 (96.7%)	82 (93.2%)	2 (66.7%)	0.678
Lobular	8 (6.6%)	1 (3.3%)	6 (6.8%)	1 (33.3%)	
**ER, % (median)**	90	90	90	80	0.414
**PR, % (median)**	40	45	40	80	0.860
**Ki-67, % (median)**	30	30	30	40	0.876
**Grade**					
1	4 (3.3%)	0 (0.0%)	3 (3.4%)	1 (33.3%)	0.248
2	79 (65.3%)	20 (66.7%)	58 (65.9%)	1 (33.3%)	
3	38 (31.4%)	10 (33.3%)	27 (30.7%)	1 (33.3%)	
**Visceral metastasis**	53 (43.8%)	13 (43.3%)	40 (45.5%)	0 (0.0%)	1.000
**Bone metastasis**	87 (71.9%)	21 (70.0%)	64 (72.7%)	2 (66.7%)	0.820
**Liver metastasis**	25 (20.7%)	7 (23.3%)	18 (20.5%)	0 (0.0%)	0.794
**Lung metastasis**	30 (24.8%)	7 (23.3%)	23 (26.1%)	0 (0.0%)	0.808
**CNS metastasis**	7 (5.8%)	2 (6.7%)	5 (5.7%)	0 (0.0%)	1.000
**CDK4/6 inh. line**					
1st line	81 (66.9%)	20 (66.7%)	61 (69.3%)	0 (0.0%)	0.820
≥2nd line	40 (33.1%)	10 (33.3%)	27 (30.7%)	3 (100.0%)	
**CDK4/6 inh.**					
Ribociclib	84 (69.4%)	22 (73.3%)	61 (69.3%)	1 (33.3%)	0.820
Palbociclib	36 (29.8%)	8 (26.7%)	26 (29.5%)	2 (66.7%)	
Abemaciclib	1 (0.8%)	0 (0.0%)	1 (1.1%)	0 (0.0%)	
**Endocrine partner**					
Letrozole	84 (69.4%)	24 (80.0%)	60 (68.2%)	0 (0.0%)	0.250
Fulvestrant	37 (30.6%)	6 (20.0%)	28 (31.8%)	3 (100.0%)	

Footnote: Values are *n* (%) unless otherwise indicated. Continuous variables are presented as median (IQR). *p*-values compare BRCA1 vs. BRCA2 groups only; patients with dual BRCA1 + BRCA2 variants are shown descriptively. Abbreviations: ECOG, Eastern Cooperative Oncology Group; ER, estrogen receptor; PR, progesterone receptor; CNS, central nervous system.

**Table 2:** Efficacy, survival and safety outcomes according to BRCA status.

	All (*n* = 121)	BRCA1 (*n* = 30)	BRCA2 (*n* = 88)	BRCA1 + 2 (*n* = 3)	*p* *
**Best response**					
CR	15 (12.4%)	6 (20.0%)	9 (10.2%)	0 (0%)	0.38
PR	69 (57.0%)	17 (56.7%)	50 (56.8%)	2 (66.7%)	
SD	16 (13.2%)	4 (13.3%)	11 (12.5%)	1 (33.3%)	
PD	21 (17.4%)	3 (10.0%)	18 (20.5%)	0 (0%)	
**ORR (CR + PR)**	84 (69.4%)	23 (76.7%)	59 (67.0%)	2 (66.7%)	0.37
**CBR (CR + PR + SD)**	100 (82.6%)	27 (90.0%)	70 (79.5%)	3 (100%)	0.27
**Progression, *n* (%)**	86 (71.1%)	17 (56.7%)	66 (75.0%)	3 (100%)	—
**Median PFS, months (95% CI)**	17.0 (14.1–19.9)	25.0 (15.9–34.1)	14.0 (10.3–17.7)	6.0 (NA)	0.135
**24-month PFS rate, %**	33%	51%	29%	0%	—
**Death, *n* (%)**	42 (34.7%)	11 (36.7%)	28 (31.8%)	3 (100%)	—
**Median OS, months (95% CI)**	47.0 (38.2–55.8)	57.0 (23.1–90.9)	49.0 (NA)	11.0 (3.0–19.0)	0.520
**24-month OS rate, %**	78%	72%	82%	0%	—
**Dose reduction due to toxicity, *n* (%)**	20 (16.5%)	3 (10.0%)	17 (19.3%)	0 (0%)	0.40
**Discontinuation due to toxicity, *n* (%)**	3 (2.5%)	0 (0%)	3 (3.4%)	0 (0%)	0.57

Footnote: * *p*-values compare BRCA1 and BRCA2 carriers; the dual BRCA1/2 subgroup (*n* = 3) is shown descriptively and was not included in statistical testing. Time-to-event outcomes (PFS and OS) were compared using the log-rank test, and categorical variables using Pearson’s chi-square or Fisher’s exact test as appropriate. Abbreviations: CBR, clinical benefit rate; CI, confidence interval; CR, complete response; ORR, objective response rate; OS, overall survival; PD, progressive disease; PFS, progression-free survival; PR, partial response; SD, stable disease.

**Table 3:** Univariable and multivariable Cox regression analysis for PFS.

Variable	Univariable HR (95% CI)	*p*	Multivariable HR (95% CI)	*p*
Age (per year)	0.995 (0.977–1.013)	0.560	0.987 (0.968–1.007)	0.206
**ECOG ≥ 1 vs. 0**	**1.789 (1.169–2.736)**	**0.007**	**1.846 (1.159–2.940)**	**0.010**
BRCA2 vs. BRCA1	1.500 (0.875–2.546)	0.137	1.246 (0.714–2.176)	0.439
Liver metastasis	1.748 (1.057–2.891)	0.029	1.366 (0.798–2.340)	0.255
De novo metastatic disease	0.646 (0.416–1.003)	0.051	0.817 (0.503–1.327)	0.415
**Fulvestrant vs. letrozole**	**2.031 (1.285–3.211)**	**0.002**	**1.735 (1.024–2.941)**	**0.041**
Bone metastasis	1.206 (0.734–1.983)	0.459	—	—
Lung metastasis	0.925 (0.564–1.518)	0.758	—	—
Brain metastasis	1.623 (0.705–3.736)	0.255	—	—
Visceral metastasis	1.291 (0.836–1.992)	0.249	—	—
CDK4/6 inh.	0.776 (0.485–1.241)	0.290	—	—
Line of CDK4/6 inh. use	1.302 (0.824–2.058)	0.258	—	—
Prior CT before CDK4/6 inh.	1.288 (0.804–2.064)	0.292	—	—
Grade ≥ 3 toxicity	0.747 (0.426–1.308)	0.307	—	—
Dose reduction	0.827 (0.465–1.471)	0.517	—	—

Footnote: Variables with *p* < 0.10 in univariable analysis and/or considered clinically relevant a priori (age, BRCA type) were included in the multivariable Cox proportional hazards model. Bold indicates variables that remained statistically significant in the multivariable analysis. The multivariable model was adjusted for age, ECOG performance status (0 vs. ≥1), BRCA mutation type (BRCA1 vs. BRCA2), presence of liver metastasis, de novo metastatic disease, and endocrine partner (letrozole vs. fulvestrant). Abbreviations: PFS, progression-free survival; HR, hazard ratio; CI, confidence interval; ECOG, Eastern Cooperative Oncology Group; CT, chemotherapy.

**Table 4:** Univariable and multivariable Cox regression analysis for OS.

Variable	Univariable HR (95% CI)	*p*	Multivariable HR (95% CI)	*p*
Age (per year)	0.99 (0.96–1.01)	0.341	—	—
ECOG ≥ 1 vs. 0	1.85 (0.98–3.49)	0.059	1.705 (0.900–3.233)	0.102
BRCA2 vs. BRCA1	0.79 (0.39–1.60)	0.520	—	—
De novo metastatic disease	0.84 (0.44–1.59)	0.584	—	—
Bone metastasis	1.46 (0.66–3.19)	0.350	—	—
Liver metastasis	0.78 (0.34–1.77)	0.556	—	—
Lung metastasis	0.71 (0.33–1.49)	0.358	—	—
Brain metastasis	2.27 (0.69–7.42)	0.177	—	—
CDK4/6 inh.	0.59 (0.31–1.11)	0.104	0.708 (0.371–1.353)	0.296
**Fulvestrant vs. letrozole**	**2.53 (1.35–4.74)**	**0.004**	**2.389 (1.257–4.540)**	**0.008**
Grade ≥ 3 toxicity	1.01 (0.46–2.20)	0.983	—	—
Dose reduction	0.83 (0.47–1.47)	0.517	—	—

Footnote: Variables with *p* < 0.10 in univariate analysis were considered for multivariate modeling. Bold indicates variables that remained statistically significant in the multivariable analysis. Multivariate model included ECOG, CDK4/6 inhibitor type, and endocrine partner. The final model was statistically significant (χ^2^ = 12.6, *p* = 0.006). Abbreviations: HR: hazard ratio; CI: confidence interval; OS: overall survival; ECOG: Eastern Cooperative Oncology Group.

## Data Availability

The datasets generated and analyzed during the current study are available from the corresponding author on reasonable request.
